# Effect of hypochlorous acid in open wound healing

**DOI:** 10.6026/9732063002001619

**Published:** 2024-11-30

**Authors:** Spurgeon Raj Jalem, Purimitla Usharani

**Affiliations:** 1 Department of Emergency medicine and critical care, Bliss Hospital, Murad Nagar Road, Mehidipatnam, Hyderabad - 500028, Telangana, India; 2 Department of Microbiology, Dr. Patnam Mahender Reddy Institute of Medical Sciences, Chevella, Ranga Reddy, Telangana, India

**Keywords:** Hypochlorous acid, chronic wound, infection, co-morbidities

## Abstract

Wound healing is a complex process involving multiple stages to restore the integrity and function of damaged tissues. Researchers are exploring the therapeutic potential of HOCl in treating infections and inflammatory conditions, given its efficacy in killing pathogens and modulating immune responses. The present study is a prospective study aimed to know the wound healing nature of HOCl on open wounds in patients attending out-patient department. The study was conducted for a period of one year Jan 2023 to Jan 2024 at Bliss Hospital, Hyderabad, Telangana district, India. Hypochlorous acid (Vida solutions, Pvt. Ltd) was used to irrigate the open wound. Among the 10 reported cases, three were females and seven were males. Most of the cases were reported from middle-age and old-age persons except one paediatric case of age 3 years a female girl. A male patient of age 72 years with co-morbidities diabetes and hypertension suffering with chronic wound on left leg for a period of 35 years because of thorn prick injury. The complete wound healing took nearly 15 weeks in this patient. The wound healing takes place 8 to 15 weeks maximum in all the 10 cases and the above findings concluded that, these features lead to a stabilized HOCl solution as an ideal wound care agent.

## Background:

Wound healing is a complex process involving multiple stages to restore the integrity and function of damaged tissues [1]. The stages of wound healing involve constriction of blood vessels to reduce blood flow and aggregation of platelets to form a clot, releasing factors that initiate the healing process. The factors affecting the wound healing includes infection which prolongs the inflammation and delays the wound healing, adequate blood supply, proper moisture which balance and promotes cell migration and reduces the risk of infection, age, nutrition, co-morbidities, usage of medications like steroid, anti-coagulants [1]. Wound healing is of three types: primary intension: Wounds with clean edges (*e.g.*, surgical incisions) heal directly with minimal scarring. Secondary intension: Wounds with significant tissue loss (*e.g.*, ulcers) heal by filling in from the bottom and sides, leading to more significant scarring. Tertiary intension: Wounds left open for a period (*e.g.*, to allow drainage) and then closed later. Wound healing is a dynamic process that involves the mechanisms like hemostasis, inflammation, proliferation and maturation or remodeling [2]. Each phase is regulated by a complex interplay of cells, growth factors, cytokines and extracellular matrix components, ensuring that tissue integrity and function are restored efficiently [2]. Understanding these mechanisms is vital for developing therapies to enhance wound healing and manage chronic wounds effectively. Chronic wounds result from a complex interplay of local and systemic factors like bacterial infection, colonization by biofilm, peripheral arterial disease, venous insufficiency, diabetic neuropathy, chronic inflammation, nutritional deficiency, usage of any medications like steroids, chemotherapeutic drugs, age, co-morbidities, bedsores, immune-compromised and smoking that impair the normal healing process [3]. Effective management requires addressing the underlying causes, optimizing wound care and potentially utilizing advanced therapies such as growth factors, skin substitutes or hyperbaric oxygen therapy to promote healing [4]. Biofilms are complex communities of microorganisms that adhere to surfaces and are embedded within a self-produced matrix of extracellular polymeric substances (EPS). Biofilms consist of bacteria, fungi and sometimes viruses that are encased in a matrix composed of polysaccharides, proteins and nucleic acids. The matrix protects the microorganisms from the host immune system like phagocytosis and antimicrobial treatments. Biofilms can impede the healing process by causing persistent inflammation. They provide a reservoir for continuous infection and can lead to the development of chronic wounds [4]. The EPS matrix can hinder the penetration of antibiotics and other treatments, making infections difficult to eradicate. Staphylococcus aureus and Pseudomonas aeruginosa are two of the most common bacteria involved in biofilm formation in wounds. Other bacteria, such as Enterococcus spp. Escherichia coli and various anaerobes, can also form biofilms in wound environments. The generation of reactive oxygen species (ROS) is a vital part of the host immune defense against pathogens. Phagocytes, such as neutrophils and macrophages, produce ROS through the activation of NADPH oxidase, which converts oxygen to superoxide anion [5-6]. This superoxide is further converted to hydrogen peroxide by superoxide dismutase and in neutrophils, myeloperoxidase uses hydrogen peroxide to produce hypochlorous acid, a powerful antimicrobial agent. Additionally, hydrogen peroxide can participate in the Fenton reaction to produce hydroxyl radicals, which are highly damaging to pathogens. ROS directly kill pathogens by damaging their membranes, proteins and DNA. They also act as signaling molecules, modulating immune responses and inflammatory reactions. Furthermore, ROS can induce autophagy and apoptosis, aiding in the removal of infected cells [5- 6]. However, excessive ROS production can cause oxidative stress and damage to host tissues, necessitating a balance maintained by antioxidant systems. These systems include enzymes like catalase and glutathione peroxidase, which neutralize excess ROS to protect host cells. Hypochlorous acid (HOCl) is a critical component of the innate immune system with several important roles in defending the body against pathogens. HOCl is highly effective at killing a wide range of pathogens, including bacteria, viruses, fungi and protozoa. It achieves this by causing oxidative damage to microbial cell walls, membranes, proteins and nucleic acids, leading to cell lysis and death. Its broad-spectrum activity makes it a crucial agent in the rapid elimination of diverse pathogens encountered by the immune system [6]. Researchers are exploring the therapeutic potential of HOCl in treating infections and inflammatory conditions, given its efficacy in killing pathogens and modulating immune responses. Since the majority of topical antiseptics have a cytotoxic effect that hinders wound healing and biofilm formation is one of the main causes of chronic wound infections, therapeutic strategies against biofilm that have high microbial eradication and good wound healing effects will lower patient morbidity and death rates as well as the associated financial burden [6]. Therefore, it is of interest to report the wound healing nature of HOCl on open wounds in OP patients attending a tertiary care Hospital.

## Materials and Methods:

Study design: It is a prospective study.

Study period: The study was conducted for a period of one year Jan 2023 to Jan 2024.

Study place: The study was conducted at Bliss Hospital, Hyderabad, Telangana district, India.

Informed consent: The study was conducted after taking the informed consent from all the ten patients.

Ethics clearance: The study was conducted after obtaining the Ethics clearance from the hospital.

## Methodology:

Hypochlorous acid (Vida solutions, Pvt. Ltd) was used to irrigate the open wound. It was in a ready-to-use form and thus did not require mixing or dilution with water or saline. We mixed 2 parts of HOCl with 1 part of Honey. Honey helps wound moistening and helps holding the HOCl on the surface of the wound bed. Dry gauze was then used to wipe the excess fluid out of the wound to prevent skin maceration. The hypochlorous acid was left inside the cavity, which was not washed with normal saline. Each patient was advised to apply thrice a day. The patients were rated the pain as 0 to 5/10 using a visual analogue scale.

## Results:

Table 1 showed all the demographic and wound history of ten cases. Among the 10 reported cases, three were females and seven were males. Most of the cases were reported from middle-age and old-age persons except one paediatric case of age 3 years a female girl have a wound on the backside of the trunk for a period of 3 weeks suffering with scabies. Another male patient of age 72 years with co-morbidities diabetes and hypertension suffering with chronic wound on left leg for a period of 35 years because of thorn prick injury. The wound healing took nearly 15 weeks in this patient. A patient of 65 male diabetic since 18 years lost his great toe of right foot due to diabetic foot which developed 20 days ago. He had surgical wound that is not responding to regular medical wound management, wound is always with continual infection and pus. He was counseled for total amputation of the foot. As last resort he was introduced to HOCl treatment which controlled infection and given hope for no amputation. Eventually in 15 weeks wound is almost closed without any skin grafting involved. HOCl helped the patient not to lose the right foot. The infection had cleared within 3 to 8 weeks in almost all of the cases. The size and the volume of the wounds were reduced by more than 95% after 6 weeks and final wound closure was achieved after 8 to 15 weeks in majority of the cases. The fever and the pain were completely subsided in all the ten cases.

## Discussion:

Chronic wounds pose significant challenges to the health care facilities as they enhance the mortality and morbidity rates globally. Several risk factors are involving in the development of chronic wounds like patient co-morbidities diabetes mellitus, peripheral vascular diseases, chronic venous insufficiency, age, immune status, pressure ulcerations *etc.* Two major elements are involved in the progression of wound healing clearance of infection and cellular interaction. The chronic wounds are generally contaminated by the patient's own skin microbial flora, external environment *etc.* The microorganisms which colonize the wound generally produce biofilms which delays the wound healing process and make the microorganisms multidrug resistant. In the present study we aimed to know the wound healing nature of HOCl on open wounds in OP patients attending a tertiary care Hospital. We treated 10 chronic wound cases with hypochlorous acid solution (HOCl) Among the 10 reported cases, three were females and seven were males. Most of the cases were reported from middle-age and old-age persons except one paediatric case of age 3 years a female girl. A male patient of age 72 years with co-morbidities diabetes and hypertension suffering with chronic wound on left leg for a period of 35 years because of thorn prick injury. The complete wound healing took nearly 15 weeks in this patient. The wound healing takes place 8 to 15 weeks maximum in all the 10 cases and the similar study was done by Serhan *et al.* (2014) [6] who determined the stabilized hypochlorous acid solution (HOCl) affected the rate at which damaged fibroblasts and keratinocytes migrated, as well as the production of biofilms and their antibacterial efficacy against commonly isolated microorganisms. Within 0 minutes, all bacteria were eliminated; the precise death period was 12 seconds. For both clinical isolates and common bacteria, the effective dose for biofilm impairment varied from 1/32 to 1/16. For every microbe, the effects of microbicides in the biofilm and anti-biofilm concentrations were the same. When compared to povidone iodine alone, the stabilized HOCl solution demonstrated dose-dependent positive effects on fibroblast and keratinocyte migration. These characteristics make a stabilized HOCl solution the perfect agent for wound treatment. Apinut *et al.* (2020) [7] published a case report and the results revealed that; immune-compromised patient with a horseshoe perianal abscess was selected to represent a heavy infection in cavity wounds. In their research, they used diluted povidone-iodine to lavage the wounds, but the fever persisted and the irrigation was painful. Hypochlorous acid was then used to irrigate the wound and within few days the fever was completely subsided Time to final wound closure was 10 weeks where as in the present study the complete wound healing took 8 to 15 weeks. These cases illustrate the effectiveness of hypochlorous acid in dealing with infection in wound cavities. A similar study by Robsonused *et al.* (2007) used stabilized solution of hypochlorous acid NVC-101 to decrease the bacterial bio burden in tissues which inhibits the bacterial infection and helps in the wound healing [8].

## Conclusion:

The above findings concluded that, these features lead to a stabilized HOCl solution as an ideal wound care agent.

## Figures and Tables

**Table 1:** Patient demographic and clinical details

**S.no**	**Age**	**Sex**	**Co-morbidities**	**Wound site**	**Occurrence of wound**	**Chronic wound period**
1	65	M	Diabetes	Right foot	Diabetic foot	4 months
2	39	F	No	Both legs	Psoriasis	5 years
3	52	M	-	Wound on right leg	Snake bite	19 years
4	72	M	-	Right leg Great toe	Thorn prick injury	35 years
5	3	F	-	Wound on backside of the trunk	Scabies	3 weeks
6	65	F	-	Multiple wounds on right hand	Fungal infection	2 years
7	70	M	Hypertension	Right leg	Venous ulcer	4 years
8	35	M	-	Backside of the neck and shoulder	Psoriasis	3 years
9	65	M	Diabetes and Hypertension	Both legs	Venous ulcer	10 years
10	55	M	Diabetes and Hypertension	Left thigh	Cellulitis	2 years
